# Damaging Behaviour and Associated Lesions in Relation to Types of Enrichment for Finisher Pigs on Commercial Farms

**DOI:** 10.3390/ani9090677

**Published:** 2019-09-12

**Authors:** Nienke van Staaveren, Alison Hanlon, Laura Ann Boyle

**Affiliations:** 1 Department of Animal Biosciences, Ontario Agricultural College, University of Guelph, Guelph, ON N1G 2W1, Canada; nvanstaa@uoguelph.ca; 2 School of Veterinary Medicine, University College Dublin, Belfield, D04 W6F6 Dublin, Ireland; alison.hanlon@ucd.ie; 3 Pig Development Department, Teagasc Moorepark, Fermoy, P61 C996 Co. Cork, Ireland

**Keywords:** enrichment, damaging behaviour, tail biting, tail lesions, ear biting, ear lesions, flank biting, flank lesions, swine

## Abstract

**Simple Summary:**

EU legislation states that all pigs must have access to material that allows them to perform investigation and manipulation activities. This reduces the risk of pigs performing damaging behaviours (e.g., tail, ear and flank biting). The aim of this study was to determine associations between damaging behaviours performed by finisher pigs, the related lesions and the use of different types of enrichment. Finisher pigs were observed on 31 commercial pig farms in Ireland and the number of pigs affected by tail, ear and flank lesions as well as all occurrences of damaging behaviour (tail-, ear- and flank-directed behaviour) were recorded. The type (chain, plastic or wood) of enrichment provided was noted; chains were the most common (41.4% of farms), followed by plastic (37.9%) and wood (20.7%). Damaging behaviour was more frequent on farms that provided chains compared to plastic or wood, particularly tail- and flank-directed behaviour was affected. The prevalence of lesions tended to be higher on farms where chains were provided compared to wooden enrichment devices. This was due to a higher prevalence of mild tail lesions on farms using chains. Results suggest that despite chains being commonly used, they did not fulfill their role in reducing damaging behaviours and associated lesions in finisher pigs.

**Abstract:**

EU legislation states that all pigs must have access to material that allows them to perform investigation and manipulation activities, thereby reducing the risk of pigs performing damaging behaviours (e.g., tail, ear and flank biting). We aimed to determine associations between damaging behaviours performed by finisher pigs, the related lesions and the use of different types of enrichment. Six randomly selected pens of finisher pigs were observed for 10 min each on 31 commercial pig farms in Ireland. All pigs were counted and the number of pigs affected by tail, ear and flank lesions was recorded. During the last 5 min, all occurrences of damaging behaviour (tail-, ear- and flank-directed behaviour) were recorded. The type (chain, plastic or wood) and number of accessible enrichment objects/pen was recorded. Chains were the most common (41.4% of farms), followed by plastic (37.9%) and wood (20.7%). Damaging behaviour was more frequent on farms that provided chains compared to plastic or wood. Farms with chains were associated with a higher frequency of flank-directed behaviour and tended to be associated with a higher frequency of tail-directed behaviour compared to farms that provided plastic devices. The prevalence of lesions tended to be higher on farms where chains were provided compared to wooden enrichment devices, mostly driven by a difference in the prevalence of mild tail lesions. Results support expert opinions that despite being commonly used, chains did not fulfill a role in reducing damaging behaviours and associated lesions in finisher pigs compared to other forms of enrichment.

## 1. Introduction

EC Directive 2008/120 requires that all pigs have access to proper investigation and manipulation materials, such as straw, hay, wood, sawdust, mushroom compost, peat or a mixture of such [1]. While these materials contain many of the characteristics preferred by pigs (i.e., ’ingestible’, ‘odorous’, ‘chewable’, ‘deformable’ and ‘destructible’), they are not always considered a practical form of environmental enrichment—especially in (partly) slatted floor systems commonly used in Europe [2,3,4]. Additionally, there is ambiguity about what qualifies as ‘proper investigation and manipulation materials’ such that farmers, consumers and animal welfare experts have different perceptions as to what constitutes appropriate enrichment for pigs [5,6,7]. Therefore, it is not surprising that in practice, enrichment is often not provided or that it is unsuitable or inadequate in many EU member states [8,9]. Further guidance was developed in 2016 to provide recommendations on how to apply the Council Directive 2008/120 as well as best practices or enrichment provision [10,11].

There is a lot of research on the effects of different forms of environmental enrichment, including alternative systems, straw-based systems and different point-source enrichment objects (e.g., hanging toys or objects), on pig behaviour, health and physiology, and performance [3,12,13,14]. In general, environmental enrichment provides pigs with opportunities to express explorative behaviour and its presence aids in reducing damaging behaviours, such as tail biting, compared to when pigs are housed in barren environments [4,12,14]. Pigs that perform tail biting are also likely to perform other forms of damaging behaviour such as ear biting [15]. Awareness of ear biting as a potential welfare issue is increasing and several studies report higher levels of ear lesions and/or biting than tail lesions and/or biting [16,17,18]. While environmental enrichment can reduce both tail and ear biting in pigs to some extent [19], much of the focus has been on tail biting, with the majority of studies being conducted under experimental conditions rather than on commercial farms [12,14].

This paper aimed to describe associations between damaging behaviours (i.e., tail-, ear-, and flank-directed behaviour) and the related lesions, and the type of enrichment provided to finisher pigs on commercial farrow-to-finish farms.

## 2. Materials and Methods

A cross-sectional welfare assessment was carried out on 31 farrow-to-finish Irish pig farms as part of a larger study [18,20]. All commercial farms practiced routine tail-docking and details regarding housing and management are described in van Staaveren et al. [18]. The assessment was conducted as described in van Staaveren et al. [18]. In brief, six pens of finisher pigs were selected using proportionate stratified sampling to account for different numbers of pigs in each house. Pigs were observed for a 10-min period from outside of each pen. All pigs were counted and the number of pigs affected by different lesions associated with damaging behaviour was recorded. Tail lesions were scored as either the presence of mild tail lesions (evidence of injuries caused by chewing but no evidence of swelling) or the presence of severe tail lesions (bloody, swollen and/or amputated tail) [21,22]. Similarly, the presence of mild and severe ear lesions and flank lesions were noted [16]. During the last 5 min of the observation, all occurrences of damaging behaviour (tail-, ear-, and flank-directed behaviour) were recorded. The percentage of pigs affected by all lesions combined as well as tail, ear and flank lesions was calculated for each pen. Likewise, the frequency of all damaging behaviours (combined and separate) performed per pig during the 5-min observation period was calculated by dividing the total number of tail-, ear-, and flank-directed behaviours by the number of pigs in the pen. Average values of lesion prevalence and behaviour frequency were calculated over the six observed pens for each farm. Spearman rank correlations between the observed behaviour and the prevalence of associated lesions were calculated (PROC CORR).

The type and number of accessible enrichment objects provided in each pen was recorded. Enrichment type was classified as either being a chain-type (metal chains), plastic-type (plastic, PVC or rubber objects), wood-type (planks of timber or pieces of wood a t the end of chains) or rope-type enrichment (natural fibre or artificial ropes of varying length). The main type of enrichment provided was determined for each farm, however, two farms provided a variety of the different enrichment types and were excluded from analysis. The effect of enrichment type and the average number of enrichment devices provided per pig on the prevalence of tail, ear and flank lesions and frequency of damaging behaviour was evaluated using a generalized linear mixed model (PROC GLIMMIX). Due to the low prevalence of severe tail, ear and flank lesions, we also considered the presence of these severe lesions on-farm as a binary outcome.

A Tukey-Kramer adjustment was used to account for multiple comparisons. All statistical procedures were conducted using SAS V9.4 (SAS Inst. Inc., Cary, NC, USA) [23]. The assumptions of normally distributed residuals and homogeneity of variance were examined graphically with the use of QQ plots. Data were transformed where necessary. Statistical significance was considered at *p* < 0.05 and tendencies were reported when 0.05 ≤ *p* ≤ 0.1. Values are presented as (back transformed) least square (LS) means ± SE, unless stated otherwise.

## 3. Results

The complete results of the welfare assessment are presented in van Staaveren et al. [18]. Overall, the average prevalence of lesions was 20.4 ± 1.62% (range: 5.2–36.8%) while the frequency of damaging behaviour was 0.15 ± 0.01 occurrences/pig/5 min (range: 0.02–0.30). The average prevalence and range of tail, ear and flank lesions and the frequency of damaging behaviours performed by finisher pigs are presented in Table 1. The prevalence of mild and severe flank lesions were positively correlated, however, none of the other lesion prevalences were correlated (Table 2). Tail-directed behaviour was not correlated with ear-directed behaviour (r = 0.09, *p* = 0.6489) or flank-directed behaviour (r = 0.08, *p* = 0.6770), and neither was ear-directed behaviour correlated with flank-directed behaviour (r = −0.13, *p* = 0.4480).

Tail-directed behaviour and ear-directed behaviour were positively correlated with the prevalence of tail and ear lesions, respectively (Table 3). Flank-directed behaviours were not correlated with the prevalence of flank lesions (Table 3)

### 3.1. Associations Between Enrichment Type and Damaging Behaviour

The majority of farms provided chains as enrichment (41.4%), followed by plastic-type (37.9%) and wood-type (20.7%); none of the farms provided rope-type enrichment (Table 4). The average number of pigs (F_2,26_ = 5.81, *p* = 0.0082) and enrichment objects per pen (F_2,26_ = 6.15, *p* = 0.0065) was highest on farms that provided wood-type enrichment (Table 4), however, the average number of enrichments provided per pig did not differ between farms that provided chains, plastic or wood-type enrichments (F_2,26_ = 2.63, *p* = 0.1000, Table 4).

**Figure 1:**
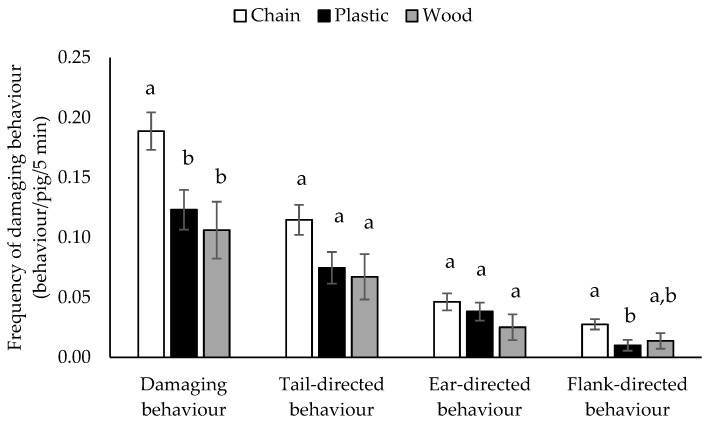
Frequency (LS means ± SE) of all damaging behaviours combined and tail-, ear-, and flank-directed behaviours observed during 5 min (behaviour/pig/5 min) in finisher pigs on 29 farms that provided different types of enrichment (chains, plastic or wooden devices). Different superscript letters indicate significant differences *p* < 0.05.

The amount of damaging behaviour observed during 5 min observation per pen differed depending on the type of enrichment provided (F_2,25_ = 6.17, *p* = 0.0066, Figure 1). A higher amount of damaging behaviour was observed in farms where chains were provided compared to farms where plastic (t_25_ = 2.91, *p* = 0.0200) or wooden (t_25_ = 2.86, *p* = 0.0222) enrichment devices were provided. There was no difference between plastic or wooden enrichment devices in terms of their association with the frequency of damaging behaviour (t_25_ = 0.57, *p* = 0.8383). The number of enrichment devices provided per pig was not associated with the amount of damaging behaviour observed (F_1,25_ = 0.02, *p* = 0.8808).

The amount of tail-directed behaviour observed differed depending on the type of enrichment provided (F_2,25_ = 3.41, *p* = 0.0488, Figure 1), however, the amount of tail-directed behaviour tended to differ only between chain and plastic enrichment devices (t_25_ = 2.22, *p* = 0.0869). The number of enrichment devices provided per pig was not associated with the amount of tail-directed behaviour observed (F_1,25_ = 0.38, *p* = 0.5459).

Ear-directed behaviour was not affected by providing different types of enrichment (F_2,25_ = 1.35, *p* = 0.2778, Figure 1) and the number of enrichment devices provided per pig was not associated with the amount of ear-directed behaviour observed (F_1,25_ = 0.57, *p* = 0.4578).

Comparing all types of enrichment devices (F_2,25_ = 4.28, *p* = 0.0251, Figure 1), a higher frequency of flank-directed behaviour was observed on farms where chains were provided compared to farms where plastic enrichment devices were provided (t_25_ = 2.81, *p* = 0.0247), while there was no difference with farms that provided wooden enrichment devices (t_25_ = 1.74, *p* = 0.2111). The same amount of flank-directed behaviour was observed when plastic- or wooden-type enrichments were provided (t_25_ = −0.45, *p* = 0.8962). Additionally, the number of enrichment devices provided per pig did not affect the frequency of flank-directed behaviour (F_1,25_ = 0.00, *p* = 0.9911).

### 3.2. Associations Between Enrichment Type and Tail, Ear and Flank Lesions

There was a tendency for enrichment type to affect the overall prevalence of lesions (F_2,25_ = 2.79, *p* = 0.0803, Table 5). Farms where chains were provided tended to have a higher prevalence of lesions than those where wood was provided (t_25_ = 2.36, *p* = 0.0654). There was no difference between chains and plastic- (t_25_ = 0.74, *p* = 0.7407), or plastic- and wooden-type enrichments (t_25_ = 1.72, *p* = 0.2163). The number of enrichment devices provided per pig was not associated with a higher prevalence of lesions (F_1,25_ = 0.08, *p* = 0.7747).

Considering the different type of lesions, the prevalence of mild tail lesions differed between the enrichment types (F_2,25_ = 4.43, *p* = 0.0226, Table 5). The prevalence of mild tail lesions was significantly lower on farms where wood was provided compared to farms where chains were the main type of enrichment (t_25_ = 2.92, *p* = 0.0192). Farms that provided plastic objects were intermediate. The prevalence of severe tail lesions was not associated with a certain type of enrichment (F_2,25_ = 1.98, *p* = 0.1595, Table 5) and neither was the presence of severe tail lesions on farms (F_2,25_ = 0.17, *p* = 0.8457). An increase in the number of enrichment devices provided per pig tended to be associated with a higher prevalence of severe tail lesions (enrichment type: +22.7 ± 12.13 increase, F_1,25_ = 3.51, *p* = 0.0727).

The type of enrichment was not associated with differences in the prevalence of ear lesions (F_2,25_ = 1.27, *p* = 0.2976), regardless of whether these were mild (F_2,25_ = 1.55, *p* = 0.2315) or severe (F_2,25_ = 1.17, *p* = 0.3283) lesions (Table 5). Additionally, the presence of severe ear lesions was not influenced by the type of enrichment provided (F_2,25_ = 0.84, *p* = 0.4440). However, a higher number of enrichment devices provided per pig was associated with a lower prevalence of mild ear lesions (enrichment type: −19.8 ± 8.85, F_1,25_ = 4.99, *p* = 0.0346).

The prevalence of flank lesions (F_2,25_ = 0.80, *p* = 0.4616), whether mild (F_2,25_ = 0.82, *p* = 0.4515) or severe (F_2,25_ = 0.60, *p* = 0.5556), did not differ between farms that provided chain-, plastic- or wooden enrichment devices (Table 3). The type of enrichment was not associated with the presence of pigs with severe flank lesions on-farm (F_2,25_ = 1.64, *p* = 0.2149). Furthermore, the number of enrichment devices provided per pig was not correlated with the prevalence of any of the flank lesions (mild: F_1,25_ = 0.02, *p* = 0.8888; severe: F_1,25_ = 0.03, *p* = 0.8552).

## 4. Discussion

This paper presents findings on environmental enrichment use on Irish farrow-to-finish pig farms, its relationship to damaging behaviours performed by pigs and to the prevalence of the associated lesions. Some limitations of this study must be acknowledged. First, these findings were part of a larger study [18,20] and investigating the effectiveness of enrichment was not the main aim. However, as a recent cross-sectional survey, it gives an overview of commonly used enrichment devices and levels of damaging behaviour performed by pigs on commercial farms with fully slatted flooring, which has not been reported previously. Our findings are in agreement with other studies reporting a high use of chains and other point-source objects as environmental enrichment on pig farms [6,7,8]. For example, farmers surveyed in the Netherlands mainly provided chains (52–63%) or hanging rubber or plastic balls (22–30%). In the current study, there was a more balanced division between chains and plastic objects, while a small number of farms provided wooden objects. The provision of hard wood is more common in the UK, which is specified under the Red Tractor assurance scheme [7]. None of the farms provided straw or other substrates as also reported by conventional Dutch pig farmers [6]. This practice seems to be more common in countries where pigs are reared with intact tails (e.g., Sweden [24,25]) and in countries where straw is more readily available (e.g., UK [26]). Straw or other substrates as well as wood meet more of the characteristics of appropriate enrichment material in that they are edible and destructible in contrast to chains and plastic-type enrichment [3,4]. This highlights that the official guidance developed by the expert working group set up by the European Commission is not adopted by the industry and the lack of enforcement of EU legislation [9,10,11].

The prevalence of tail, ear and flank lesions and the frequency of damaging behaviour was recorded in this study as part of a larger on-farm welfare assessment showing a high variability between farms [18,20]. There were no correlations between the prevalence of the different types of lesions as similarly observed in van Staaveren et al. [18], suggesting that tail-, ear-, and flank-lesions can be distinct problems and that they are not necessarily interconnected. Behaviour was only observed for a short period of time to complete the full on-farm welfare assessment [18,27], and a longer observation period may provide more insights in the performance of damaging behaviour. The short observation time could explain the lack of correlations between the frequency of damaging behaviours expressed and the prevalence of the associated lesions. Despite the short observation period, tail-, ear- and flank-directed behaviours were noted on nearly all farms which raises concern about pig welfare. The main goal of providing enrichment is to give pigs an opportunity to express species-specific behaviour and manage the expression of damaging behaviour [8]. Ranking of different enrichment materials by an expert working group classified, e.g., chains and plastic objects, as materials with marginal interest, e.g., wood or rope still as suboptimal while, e.g., straw and fodder, were considered as optimal materials [10,11]. Thus, while none of the point-source objects provided to pigs in the studied farms could be considered as optimal enrichment devices as they had only limited occupational value to pigs [8], our findings support that the provision of metal chains is unlikely to aid in managing damaging behaviour [7,9]. Chains hanging too high are considered to be the less acceptable form of enrichment, while branched chains (one long chain reaching the pen floor with additional shorter chains attached at pig height) are considered a viable alternative by pig experts, reaching close to the acceptable level [7]. It should be noted that chains provided on the studied farms typically existed of single chains (none-branched chains), but they were hanging within the pigs’ reach.

Both tail-directed and flank-directed behaviours were observed more frequently on farms that provided chains rather than plastic- or wooden enrichment devices. Furthermore, these results were partly reflected in the tail lesion prevalence, especially regarding mild tail lesions. A lower prevalence of mild tail lesions was observed in farms with plastic- or wooden enrichments, particularly wood, than when chains were provided. Additionally, we observed a lower prevalence of ear lesions when plastic- or wooden objects were provided compared to chains, though this difference was not statistically significant. While Telkänranta et al. [19] did not find differences in damaging behaviour in pigs provided with chains, polythene pipes or wooden branches, they similarly observed less pigs with injured ears or mild tail lesions when provided with wooden branches. Pigs are likely to interact differently with different wood types [28], however, in the current study, we could not determine what kind of wood was provided. Little is known about ear- and flank-directed behaviour in pigs, however the lesions associated with these behaviours can be compounded by pathogens [29,30].

With tail-directed behaviour and tail lesions, the role of enrichment is clearer and providing novel enrichment to pigs is often recommended in the event of a tail biting outbreak [4,14]. Our results suggest that chains are less effective at preventing or reducing tail-directed behaviour and therefore, more tail lesions are observed (mostly superficial lesions) with this type of enrichment. However, once a tail biting outbreak has started, enrichment type does not appear to affect the prevalence of tail lesions, as shown by a lack of differences between the different enrichment types in terms of the prevalence of severe tail lesions. This is likely due to the difficulty in controlling a tail biting outbreak once it has started if the intervention is not implemented early enough [31]. Whilst there was a low prevalence of severe tail lesions in the study, this could also explain the finding that a higher number of enrichment objects provided per pig was associated with a higher prevalence of severe tail lesions. This finding was contrary to what we expected but due to the cross-sectional nature of the study, we cannot ascertain when enrichment was provided, and whether or not farmers added more enrichment devices in an attempt to control a tail biting outbreak. A survey of Finnish pig farmers with undocked pigs attached less importance to adding enrichment objects to a pen when tail biting had started compared to other intervention measures (e.g., removing biter) [32]. However, we did not ask farmers in the current study about their attitudes towards environmental enrichment, nor did we question their underlying reasons behind their current environmental enrichment management strategy (e.g., type, number of enrichment objects provided).

Finally, the role of farmer perceptions of animal welfare and the effectiveness of enrichment, and general management cannot be excluded. Farmers’ attitudes and beliefs regarding animal welfare also informs their decision making process and influences a farmer’s readiness to implement certain management practices [33,34]. Bock and van Huik [34], who reported on European pig farmer attitudes and behaviour, found that farmers that participate in quality-assurance schemes and organic or specific welfare schemes have different definitions of animal welfare and this influenced their farming style and acceptability of further welfare regulations. Similarly, in a survey amongst Dutch pig farmers, conventional and organic pig farmers both considered tail biting to be an important welfare issue but conventional farmers considered enrichment to be of less importance and considered tail docking necessary compared to organic pig farmers [6]. Additionally, farmers can differ in what level of tail biting they consider acceptable [32]. It is also possible that the differences between farms where plastic- or wooden enrichment objects are provided as opposed to chain-type enrichment was caused by a difference in farmer attitude and general management. However, to the authors’ knowledge, no studies have looked at differences in farmer attitudes, management styles and the different types of enrichment provided to pigs. Nevertheless, given the high number of risk factors for damaging behaviour [4] it cannot be ruled out that slightly better enrichment devices (plastic and wood) were simply proxies for better management of pig health and welfare in other areas.

## 5. Conclusions

This study aimed to determine associations between damaging behaviours performed by finisher pigs, the related lesions and the use of different types of enrichment. Observations on 31 commercial farrow-to-finish farms suggest that point-source objects, such as chains, plastic objects and wood, are commonly used. Higher frequencies of damaging behaviour (specifically tail- and flank-directed behaviour), and a higher prevalence of mild tail lesions were found on farms where chains were provided. These results support the need for the development of standards and education on suitable environmental enrichment material for finisher pigs.

## Figures and Tables

**Table 1:** Average prevalence (mean ± SE) and range (min–max) of lesions and average frequency of damaging behaviours/pig/5 min observation expressed by finisher pigs on 31 farrow-to-finish pig farms based on raw data.

Variable	Mean	Min	Max
Lesions (%)			
Mild tail lesions	9.2 ± 0.73	3.53	20.85
Severe tail lesions	1.8 ± 0.36	0.00	6.20
Mild ear lesions	1.4 ± 0.33	0.00	5.16
Severe ear lesions	6.2 ± 1.14	0.00	22.33
Mild flank lesions	0.3 ± 0.17	0.00	5.10
Severe flank lesions	1.9 ± 0.39	0.00	6.82
Damaging Behaviour (occurrences/pig/5 min)			
Tail-directed	0.09 ± 0.008	0.008	0.196
Ear-directed	0.04 ± 0.004	0.00	0.098
Flank-directed	0.02 ± 0.003	0.00	0.063

**Table 2:** Correlations between the prevalence of finisher pigs with associated tail-, ear-, and flank-lesions on 31 farrow-to-finish pig farms. *p*-values are given in brackets.

Variable	1	2	3	4	5	6
Lesions (%)						
Mild tail lesions (1)						
Severe tail lesions (2)	−0.00 (0.9842)					
Mild ear lesions (3)	+0.25 (0.1677)	−0.13 (0.4995)				
Severe ear lesions (4)	+0.12 (0.5309)	+0.30 (0.1014)	+0.30 (0.1033)			
Mild flank lesions (5)	−0.14 (0.4515)	−0.03 (0.8777)	+0.20 (0.2757)	+0.03 (0.8651)		
Severe flank lesions (6)	+0.16 (0.3815)	0.00 (0.9955)	+0.13 (0.4920)	+0.22 (0.2416)	+0.46 (0.0097)	

**Table 3:** Correlations between damaging behaviours observed during a 5 min period and the prevalence of finisher pigs with associated lesions on 31 farrow-to-finish pig farms.

Variable	Correlation	*p*-Value
Tail-directed behaviour		
Mild tail lesions	+0.51	0.0034
Severe tail lesions	+0.25	0.1765
Ear-directed behaviour		
Mild ear lesions	−0.11	0.5456
Severe ear lesions	+0.41	0.0206
Flank-directed behaviour		
Mild flank lesions	0.05	0.7931
Severe flank lesions	0.05	0.7703

**Table 4:** Number of farms providing different types of enrichment (chain, plastic objects, wooden objects) and average number of pigs, enrichment devices and enrichment devices per pig (LS means ± SE) on 29 farrow-to-finish pig farms. Different superscript letters indicate significant differences *p* < 0.05.

Variable	Chain	Plastic	Wood
No. of farms	12	11	6
Average no. of pigs per pen	19.8 ± 2.20 ^a^	23.3 ± 2.30 ^a,b^	32.7 ± 3.12 ^b^
Average no. of enrichment devices per pen	1.0 ± 0.22 ^a^	1.1 ± 0.23 ^a^	2.3 ± 0.31 ^b^
Average no. of enrichment devices per pig	0.05 ± 0.007	0.05 ± 0.008	0.08 ± 0.010

**Table 5:** Prevalence (LS means ± SE) of lesions associated with damaging behaviours observed in finisher pigs on 29 farms that provided different types of enrichment (chains, plastic or wooden objects). Different superscript letters indicate significant differences *p* < 0.05.

Variable	Chain	Plastic	Wood
Lesions (%)			
All lesions combined	23.1 ± 2.49	20.4 ± 2.64	12.2 ± 3.77
Mild tail lesions	10.6 ± 1.20 ^a^	8.2 ± 0.99 ^a,b^	5.7 ± 0.99 ^b^
Severe tail lesions	1.3 ± 0.46	1.1 ± 0.48	2.8 ± 0.69
Mild ear lesions	1.4 ± 0.33	0.7 ± 0.36	0.6 ± 0.51
Severe ear lesions	7.4 ± 1.92	6.8 ± 2.03	2.1 ± 2.90
Mild flank lesions	2.0 ± 0.29	0.6 ± 0.30	0.05 ± 0.43
Severe flank lesions	1.9 ± 0.59	1.9 ± 0.62	0.8 ± 0.89
